# Feedback Bits Allocation for Guaranteed Bit Rate Services in Cooperative Cognitive Radio Networks

**DOI:** 10.3390/s20020469

**Published:** 2020-01-14

**Authors:** Deokhui Lee, Jaewoo So

**Affiliations:** 1 SK Telecom, Seongnam-si 13595, Korea; akirain@sogang.ac.kr; 2 Department of Electronic Engineering, Sogang University, Seoul 04107, Korea

**Keywords:** cooperative cognitive radio, feedback bits allocation, guaranteed bit rate service, limited feedback, multiple secondary cells

## Abstract

As the number of users using multimedia sharing services increases, the need to ensure the minimum data rate of wireless users increases. Meanwhile, in the cooperative cognitive radio (CR) network, it is important to provide the quality-of-services for secondary users (SUs) while satisfying the inter-network interference constraint from secondary transmitters to primary users (PUs). Under the limited feedback resource constraint, this paper proposes a feedback bits allocation scheme for the guaranteed bit rate services of SUs while satisfying the inter-network interference constraint. This paper investigates how many feedback bits between the ST and PUs are required to guarantee the minimum data rate of SUs and then proposes a feedback bits allocation scheme that maximizes the average sum rate of SUs while reducing the outage probability of SUs.

## 1. Introduction

As the number of users using multimedia sharing services such as Youtube increases, it is important to use limited spectrum resources efficiently in order to ensure the minimum data rate for wireless users. Cognitive radio (CR) efficiently use the limited spectrum by sharing the the spectrum assigned for a primary network with secondary users (SUs) [1,2]. In particular, the primary users (PUs) and SUs can transmit data at the same time in underlay CR networks.

In the underlay CR network, it is important to keep the inter-network interference from the secondary network to the primary network below a certain value while providing the quality-of-services (QoS) for SUs [3,4]. The power control and beamforming in CR networks is a promising technique that suppresses the inter-network interference, where the secondary transmitter (ST) needs to obtain the channel direction information (CDI) between the ST and PUs through the cooperative feedback from PUs, which results in the increase of the feedback overhead [5,6,7,8]. In the practical CR network, the ST has the imperfect CDI because of the quantization procedure due to the limited feedback bits. However, the work of [5,7] assumed the perfect CDI at the ST for using the transmit beamforming vector. Other researchers of [4,9,10,11,12] have endeavored to increase the sum rate of SUs in the CR network with the limited number of feedback bits. However, they failed to provide the guaranteed bit rate (GBR) services for SUs.

Many researchers have focused on providing the QoS for the secondary users [8,13,14,15,16]. The work of [8] designed the power control and beamforming in CR networks to increase the sum rate of SUs while providing the minimum signal-to-interference-plus-noise ratio (SINR) for SUs. The work of [13,14] proposed a resource allocation scheme that meets the delay requirements of SUs in CR networks. The work of [15] proposed a joint admission control and packet scheduling scheme that reduces the outage probability of the streaming services in an ad hoc CR network with streaming traffic and non-real-time traffic. The work of [16] developed a robust distributed power control algorithm to provide the QoS requirements for both PUs and SUs. However, the previous studies of [8,13,14,15,16] did not consider the beamforming technique with limited feedback which is an outstanding technique to mitigate the interference while increasing the sum rate. The work of [17] adaptively allocated the feedback bits and controlled the transmit power at the same time when the total number of feedback bits is fixed in the cooperative CR network, where they applied the cognitive beamforming. However, they failed to guarantee the minimum data rate for SUs. Moreover, they considered a single secondary cell in the secondary network.

This paper aims to provide the GBR services for SUs while meeting the interference constraint by using the beamforming technique in the CR network with limited feedback. This paper contributes thee things as follows: First, we investigate the number of feedback bits between the ST and PUs required to ensure the minimum bit rate of the SUs. Whereas most previous work aimed to maximize the sum rate of SUs without taking the minimum data rate of SUs into consideration. Hence, the previous schemes may cause serious starvation problems that increase the number of SUs who do not receive services or receive very low data rate. Second, we formulate two optimization problems to provide the GBR services for SUs. One is to maximize the minimum SINR in the secondary network while keeping the average inter-network interference below a certain value. We derive a suboptimal number of feedback bits between the ST and PUs to guarantee the minimum data rate of SUs. The other optimization problem is to maximize the sum rate of SUs while reducing the outage probability of SUs. To solve the optimization problem, we develop an iterative algorithm to allocate feedback bits. Third, we consider inter-cell interference (ICI) as well as inter-user interference (IUI) in multiple secondary cells of the secondary network while keeping the inter-network interference from STs to PUs below a certain value. Hence, although the results of this paper seem to be an incremental extension of the results of [17], this paper has significant differences and enhancements as follows: First, the considered system model is different. This paper considers multiple secondary cells in the secondary network while the previous work of [17] considered a single secondary cell in the secondary network. Hence, the previous work of [17] did not consider the ICI. Second, the objectives are different. This paper aims to ensure the minimum data rate for SUs while satisfying the inter-network interference constraint while the previous work of [17] aimed to maximize the sum rate of SUs without considering the minimum data rate requirement of SUs. Third, the derived results are different because of different objectives. The results show that the proposed scheme of this paper outperforms the feedback bits allocation scheme of [17] in terms of meeting the minimum data rate requirement of SUs.

The rest of this paper is organized as follows: Section 2 presents the system model and investigates the effect of the number of feedback bits on the inter-network interference. Section 3 introduces the previous feedback bits allocation scheme that maximizes the sum rate of SUs and extends the previous result to the CR network with multiple secondary cells. Section 4 presents the proposed feedback bits allocation scheme for the GBR services of SUs. Section 5 shows the numerical results and, finally, Section 6 concludes this paper.

*Notation:* The bold lower and upper case letters respectively denote column vectors and matrices. We use ${\left(\cdot \right)}^{T}$, ${\left(\cdot \right)}^{H}$, |·|, and ||·|| to denote the transpose, the conjugate transpose, the absolute value, and the norm of a vector, respectively. E[x] and $\bar{x}$ denotes the expectation of x.

## 2. System Model

### 2.1. System Description

**Figure 1:**
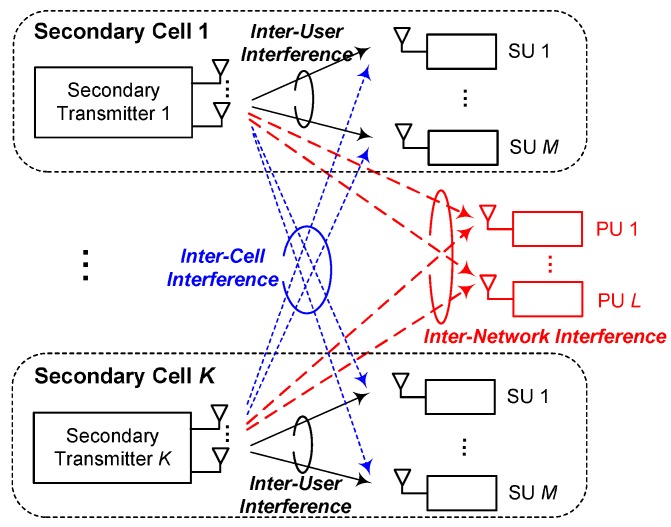
The system model.

We consider an underlay CR network, as shown in Figure 1, which consists of a primary network and multiple secondary cells. The primary network serves *L* PUs indexed by l∈L={1,⋯,L}. The secondary network consists of *K* cells. In each secondary cell, an ST with $N_{t}$ antennas serves *M* SUs with the inter-network interference constraint to PUs. Let the *k*th secondary cell be indexed by *k*, where k∈K={1,⋯,K}, and the set of served SUs in the *k*th cell be denoted by $M_{k}$, where the cardinality is $|M_{k}|=M$. The intersection of all served SUs’ sets is an empty set, i.e., $\cap_{k\in K}M_{k}=\emptyset$.

Let $B_{k,l}$ be the number of feedback bits between ST *k* and PU *l*; and $B_{k}$ is the sum of the feedback bits between ST *k* and *L* PUs, $B_{k}=\sum_{l\in L}B_{k,l}$. The total number of feedback bits is assumed to be limited to $B^{T}=\sum_{k\in K}B_{k}$. Furthermore, the total number of feedback bits per SU is limited to $b^{T}=\sum_{k\in K}b_{k,m}$, where $b_{k,m}$ is the number of feedback bits between ST *k* and SU *m*.

The ST needs to know the CDI between the ST and PUs in order to use the beamforming technique for suppressing the inter-network interference from the ST to PUs. In addition, the ST also needs to know the CDI between the ST and SUs in order to use the beamforming technique for suppressing the ICI and IUI in the secondary network. Hence, under the constraint of a limited number of feedback bits, the objective is to determine the number of feedback bits, $B_{k,l}$, between ST *k* and PU *l* and the number of feedback bits, $b_{k,m}$, between ST *k* and SU *m*.

### 2.2. Inter-Network, Inter-User, and Inter-Cell Interference

Let $P_{k}$ be the transmit power of ST *k*. Assuming the equal power transmission at the ST, the received interference signal from ST *k* to PU *l* is given by

(1) $$y_{k,l}=\sqrt{P_{k}\alpha_{k,l}}\sum_{m\in M_{k}}h_{k,l}^{H}w_{m}s_{m},$$

where $\alpha_{k,l}$ is the path loss factor from ST *k* to PU *l*; $h_{k,l}\in C^{N_{t}\times 1}$ is the channel vector from ST *k* to PU *l*; $w_{m}\in C^{N_{t}\times 1}$ is the transmit beamforming vector from ST *k* to SU $m\in M_{k}$; and $s_{m}$ denotes the transmitted symbol from ST *k* to SU *m*.

The instantaneous inter-network interference from ST *k* to PU *l* can then be expressed as follows:

(2) $$I_{k,l}=P_{k}\alpha_{k,l}\sum_{m\in M_{k}}{\left|h_{k,l}^{H}w_{m}\right|}^{2}.$$

There are two kinds of approaches on the inter-network interference constraint from the ST to the PU, the average interference constraint and the peak interference constraint. Many previous studies have considered that the average amount of interference remains below a certain value in order to increase the throughput of the secondary network [18,19]. This paper considers the average interference constraint in the underlay CR network. Hence, the average inter-network interference from ST *k* to PU *l* should be below the allowable threshold, $I_{th}$, as follows:

(3) $${\bar{I}}_{k,l}\leq I_{th},\;\forall k,\;l.$$

The received signal from ST *k* to SU *m* is given by

(4) $$\begin{array}{cccc} y_{k,m} & = & \sqrt{P_{k}\alpha_{k,m}}h_{k,m}^{H}w_{m}s_{m} & +\underset{\mathrm{inter}-\mathrm{user}\;\mathrm{interference}\;\left(\mathrm{IUI}\right)}{\underset{︸}{\sqrt{P_{k}\alpha_{k,m}}\sum_{n\in M_{k},n\neq m}h_{k,m}^{H}w_{n}s_{n}}}\\ & & & +\underset{\mathrm{inter}-\mathrm{cell}\;\mathrm{interference}\;\left(\mathrm{ICI}\right)}{\underset{︸}{\sum_{j\in K,j\neq k}\sqrt{P_{j}\alpha_{j,m}}\sum_{i\in Mj}h_{j,m}^{H}w_{i}s_{i}}}+\nu_{m},\forall m\in M_{k}, \end{array}$$

where $\alpha_{k,m}$ is the path loss factor from ST *k* to SU *m*; $h_{k,m}\in C^{N_{t}\times 1}$ is the channel vector from ST *k* to SU *m*; and $\nu_{m}$ is the noise plus interference signal from the PT to SU *m*. We approximate $\nu_{m}$ to a Gaussian random variable with zero-mean and $\sigma_{m}^{2}$ variance. All the channel elements are assumed to be drawn from independent and identically distributed (i.i.d.) complex Gaussian random variables with zero mean and unit variance. In Equation (4), the first term denotes the desired signal while the second and third terms are respectively the IUI and ICI signal.

The instantaneous SINR of SU *m* can be expressed as follows:

(5) $$\begin{array}{c} \gamma_{m}=\frac{P_{k}\alpha_{k,m}{\left|h_{k,m}^{H}w_{m}\right|}^{2}}{\sigma_{m}^{2}+I_{k,m}+I_{\bar{k},m}},\;\;\;\mathrm{for}\;m\in M_{k}, \end{array}$$

where $I_{k,m}$ and $I_{\bar{k},m}$ are respectively the IUI and the ICI, as follows:

(6) $$\begin{array}{ccc} I_{k,m} & = & P_{k}\alpha_{k,m}\sum_{n\in M_{k},n\neq m}{\left|h_{k,m}^{H}w_{n}\right|}^{2}, \end{array}$$

(7) $$\begin{array}{ccc} I_{\bar{k},m} & = & \sum_{j\in K,j\neq k}P_{j}\alpha_{j,m}\sum_{i\in Mj}{\left|h_{j,m}^{H}w_{i}\right|}^{2}. \end{array}$$

Here, $\bar{k}$ denotes the complementary indices of *k*. Please note that if the secondary network has a single cell, the ICI signal of $I_{\bar{k},m}$ is neglected in (4) and (5).

### 2.3. Average Inter-Network Interference vs. the Number of Feedback Bits

The obtained CDI at the ST is not perfect because of the quantization error due to the limited feedback bits. We investigate the average inter-network interference according to the number of feedback bits.

**Theorem** **1.** *Given $B_{k,l}$, the average inter-network interference from ST k to PU l is upper bounded by*

(8) $${\bar{I}}_{k,l}<P_{k}\alpha_{k,l}MN2^{\frac{-B_{k,l}}{N_{t}-1}},$$

*where we connote $N=N_{t}/\left(N_{t}-1\right)$ for the simplicity of notation.*

**Proof.**  According to the theorem of the random vector quantization (RVQ), we decompose the channel vector as

(9) $${\tilde{h}}_{k,l}=\sqrt{1-\kappa_{k,l}}{\hat{h}}_{k,l}+\sqrt{\kappa_{k,l}}e_{k,l},$$

where $\kappa_{k,l}$=$\sin^{2}\left(∠\left({\tilde{h}}_{k,l},{\hat{h}}_{k,l}\right)\right)$ is the amplitude of the quantization error; and $e_{k,l}$ is an i.i.d. unit vector. Because we design the transmit beamforming in order to null out the interference given the quantized CDI, we have

(10) $$|{\tilde{h}}_{k,l}^{H}w_{m}{|}^{2}=\kappa_{k,l}{\left|e_{k,l}^{H}w_{m}\right|}^{2}.$$

Using the fact that $E\left[\kappa_{k,l}\right]<2^{-B_{k,l}/\left(N_{t}-1\right)}$ and $E[|e_{k,l}^{H}w_{m}{|}^{2}]=1/\left(N_{t}-1\right)$ [20], the average inter-network interference from ST *k* to PU *l* is given by (8). □

The transmit power of an ST is limited in order to meet the average inter-network interference constraint from ST *k* to PU *l*. From (3) and (8), the transmit power of ST *k* is limited as follows:

(11) $$\begin{array}{c} P_{k}\leq \frac{I_{th}}{\alpha_{k,l}MN}2^{\frac{B_{k,l}}{N_{t}-1}},\;\forall k\in K,l\in L. \end{array}$$

Given the transmit power of $P_{k}$, we rearrange (11) in terms of the number of feedback bits between ST *k* and PU *l*, as follows:

(12) $$\begin{array}{c} B_{k,l}\geq {\left\lfloor \left(N_{t}-1\right)\log_{2}\left(\frac{P_{k}\alpha_{k,l}MN}{I_{th}}\right)\right\rfloor }^{+}. \end{array}$$

The sum of the feedback bits between ST *k* and PUs, $B_{k}=\sum_{l\in L}B_{k,l}$, can then be expressed as follows:

(13) $$\begin{array}{ccc} B_{k} & \geq & \sum_{l\in L}{\left\lfloor \left(N_{t}-1\right)\log_{2}\left(\frac{P_{k}\alpha_{k,l}MN}{I_{th}}\right)\right\rfloor }^{+} \end{array}$$

(14) $$\begin{array}{cc} = & \left(N_{t}-1\right)\left[\log_{2}{\left(\frac{P_{k}MN}{I_{th}}\right)}^{L}+\log_{2}\left(\prod_{l\in L}\alpha_{k,l}\right)\right]. \end{array}$$

Hence, after rearranging (14) again, we obtain the upper bound of the transmit power of ST *k*, as follows:

(15) $$\begin{array}{ccc} P_{k} & \leq & \frac{I_{th}}{MN}{\left(\prod_{l\in L}\alpha_{k,l}\right)}^{-1/L}2^{\frac{B_{k}}{L(N_{t}-1)}}\;≜\;{\hat{P}}_{k}. \end{array}$$

## 3. Conventional Feedback Bits Allocation for the Sum Rate Maximizing of SUs

The objective of the conventional feedback bits allocation is to maximize the sum rate of SUs given the number feedback bits [17,21,22]. However, while the previous work of [17,21,22] considers a single secondary cell, this paper considers multiple secondary cells in the cooperative CR network. Hence, given the transmit power of STs, we derive the number of feedback bits for each SU to minimize the average rate loss in an underlay CR network with multiple secondary cells. Here, the average rate loss is defined as a difference between the achievable data rate at SU $m\in M_{k}$ with the perfect CDI and with the limited feedback CDI.

According to the Theorem 1, in the limited feedback system, the average interference power at SU $m\in M_{k}$ is upper bounded as follows:

(16) $$\begin{array}{ccc} {\bar{I}}_{k,m} & \leq & P_{k}{\bar{\alpha }}_{k,m}2^{\frac{-b_{k,m}}{N_{t}-1}}, \end{array}$$

(17) $$\begin{array}{ccc} {\bar{I}}_{\bar{k},m} & \leq & \sum_{j\in K,j\neq k}P_{j}{\bar{\alpha }}_{j,m}2^{\frac{-b_{j,m}}{N_{t}-1}}. \end{array}$$

Hence, the average rate loss of SU *m* is bounded as follows:

(18) $$\begin{array}{ccc} \Delta_{m} & = & \sum_{m\in M_{k}}\log_{2}\left(\sigma_{m}^{2}+{\bar{I}}_{k,m}+{\bar{I}}_{\bar{k},m}\right)\\ & \leq & \log_{2}\left(\sigma_{m}^{2}+P_{k}{\bar{\alpha }}_{k,m}2^{\frac{-b_{k,m}}{N_{t}-1}}+\sum_{j\in K,j\neq k}P_{j}{\bar{\alpha }}_{j,m}2^{\frac{-b_{j,m}}{N_{t}-1}}\right)\\ & = & \log_{2}\left(\sigma_{m}^{2}+\sum_{j\in K}P_{j}{\bar{\alpha }}_{j,m}2^{\frac{-b_{j,m}}{N_{t}-1}}\right),\;\;\;\forall m\in M_{k}, \end{array}$$

where ${\bar{\alpha }}_{k,m}=\alpha_{k,m}\left(M-1\right)N$ and ${\bar{\alpha }}_{j,m}=\alpha_{j,m}MN$ for $m\in M_{k}$.

From (18), we can formulate the feedback bits allocation problem that minimizes the average rate loss of SUs as follows:

(19) $$\begin{array}{cc} \min_{b_{j,m}\in R^{+}} & \sum_{j\in K}P_{j}{\bar{\alpha }}_{j,m}2^{\frac{-b_{j,m}}{N_{t}-1}}\\ s.t. & \sum_{j\in K}b_{j,m}\leq b^{T},\;\forall m\in M_{k}, \end{array}$$

where $R^{+}$ denotes the set of positive real numbers for relaxing the integer $b_{k,m}$ for ∀k,m into the continuous variables. Because the optimization problem in (19) is a convex function, we can apply the convex optimization technique. In the conventional scheme of [23], the suboptimal feedback bits allocation scheme was investigated for the optimization problem in (19) as follows:

(20) $$\begin{array}{c} b_{j,m}^{*}={\left\lfloor b^{T},\frac{b^{T}}{K}+\left(N_{t}-1\right)\log_{2}\left(\frac{P_{k}{\bar{\alpha }}_{k,m}}{\prod_{j\in K}{\left(P_{j}{\bar{\alpha }}_{j,m}\right)}^{1/K}}\right)\right\rfloor }^{+}\;\;\;\;\; \end{array}$$

for $m\in M_{k}$. Hence, we find that in order to minimize the average rate loss due to the quantization error, the CDI of the link with good channel condition should be more accurate [23].

## 4. Proposed Feedback Bits Allocation for the GBR Services of SUs

### 4.1. IUI, ICI, and SU’s SINR

By substituting (20) into (16), the upper bound of the average IUI can be expressed as follows:

(21) $$\begin{array}{ccc} {\bar{I}}_{k,m} & \leq & P_{k}{\bar{\alpha }}_{k,m}2^{\frac{-b_{k,m}}{N_{t}-1}}\\ & = & 2^{\frac{-b^{T}}{K(N_{t}-1)}}\cdot \prod_{j\in K}{\left(P_{j}{\bar{\alpha }}_{j,m}\right)}^{1/K}\\ & = & \frac{I_{th}}{MN}\cdot 2^{\frac{B^{T}}{LK(N_{t}-1)}}\cdot \prod_{j\in K}{\left(\frac{{\bar{\alpha }}_{j,m}}{\prod_{l\in L}{\left(\alpha_{j,l}\right)}^{1/L}}\right)}^{1/K}\\ & ≜ & {\hat{I}}_{k,m},\;\forall m\in M_{k}, \end{array}$$

where ${\hat{I}}_{k,m}$ is the upper bound of ${\bar{I}}_{k,m}$.

Similarly, from (16), the upper bound of the average ICI is given by

(22) $$\begin{array}{ccc} {\bar{I}}_{\bar{k},m} & \leq & \sum_{j\in K,j\neq k}P_{j}{\bar{\alpha }}_{j,m}2^{\frac{-b_{j,m}}{N_{t}-1}}\\ & = & \left(K-1\right)2^{\frac{-b^{T}}{K(N_{t}-1)}}\cdot \prod_{j\in K}{\left(P_{j}{\bar{\alpha }}_{j,m}\right)}^{1/K}\\ & = & \frac{\left(K-1\right)I_{th}}{MN}2^{\frac{B^{T}-Lb^{T}}{LK(N_{t}-1)}}\cdot \prod_{j\in K}{\left(\frac{{\bar{\alpha }}_{j,m}}{\prod_{l\in L}{\left(\alpha_{j,l}\right)}^{1/L}}\right)}^{1/K}\\ & ≜ & {\hat{I}}_{\bar{k},m},\;\forall m\in M_{k}, \end{array}$$

where ${\hat{I}}_{\bar{k},m}$ is the upper bound of ${\bar{I}}_{\bar{k},m}$.

From (21) and (22), given $B^{T}$ and $b^{T}$, the closed-form of ${\hat{I}}_{k,m}$ and ${\hat{I}}_{\bar{k},m}$ can be derived taking the average inter-network interference constraint from the SU to PUs into consideration. Moreover, from (21) and (22), the SINR of SU *m* is bounded as follows:

(23) $$\begin{array}{ccc} {\bar{\gamma }}_{m} & \geq & \frac{P_{k}\alpha_{k,m}N_{t}}{\sigma_{m}^{2}+{\hat{I}}_{k,m}+{\hat{I}}_{\bar{k},m}},\;\;\forall m\in M_{k}. \end{array}$$

### 4.2. Scaling Law of Feedback Bits for GBR Services

For the GBR services of SUs, the secondary cell guarantees the average data rate of each SU, ${\bar{r}}_{m}\geq r_{\min}$, where $r_{\min}$ is the minimum data rate of the SUs. Under the constraint with ${\bar{r}}_{m}\geq r_{\min}$, from (23), we have

(24) $$\frac{P_{k}\alpha_{k,m}N_{t}}{\sigma_{m}^{2}+{\hat{I}}_{k,m}+{\hat{I}}_{\bar{k},m}}\geq \gamma_{\min},\;\forall m\in M_{k},$$

where $\gamma_{\min}=2^{r_{\min}}-1$ is the SINR constraint for satisfying the minimum data rate of SUs. Hence, from (24), the required transmit power of ST *k* is given by

(25) $$P_{k}\geq \frac{\gamma_{\min}\left(\sigma_{m}^{2}+{\hat{I}}_{k,m}+{\hat{I}}_{\bar{k},m}\right)}{\alpha_{k,m}N_{t}},\;\forall m\in M_{k}.$$

Additionally, from (15), the transmit power of ST *k* is bounded because of the average interference constraint at PUs. Hence, from (15) and (25), we obtain

(26) $$\frac{\gamma_{\min}\left(\sigma_{m}^{2}+{\hat{I}}_{k,m}+{\hat{I}}_{\bar{k},m}\right)}{\alpha_{k,m}N_{t}}\leq \frac{I_{th}}{MN}{\left(\prod_{l\in L}\alpha_{k,l}\right)}^{-1/L}2^{\frac{B_{k}}{L(N_{t}-1)}}.$$

After reformulating (26) in terms of $B_{k}$, we obtain

(27) $$\begin{array}{c} B_{k}\geq {\left\lfloor B^{T},\;\;\;L\left(N_{t}-1\right)\log_{2}\left\{\frac{\gamma_{\min}M\left(\sigma_{m}^{2}+{\hat{I}}_{k,m}+{\hat{I}}_{\bar{k},m}\right)}{I_{th}\alpha_{k,m}\left(N_{t}-1\right)}{\left(\prod_{l\in L}\alpha_{k,l}\right)}^{1/L}\right\}\right\rfloor }^{+}≜{\tilde{B}}_{k,m}, \end{array}$$

where the scaled feedback bits is derived to guarantee the minimum data rate of SU $m\in M_{k}$ taking the average inter-network interference constraint at PUs into consideration. Accordingly, to guarantee the minimum data rate of SUs served by ST *k*, the suboptimal number of feedback bits of ST *k* is given by

(28) $$\begin{array}{c} B_{k}^{*}=\max_{m\in M_{k}}{\tilde{B}}_{k,m},\;\;\forall k, \end{array}$$

where ${\tilde{B}}_{k,m}$ is obtained from (27).

### 4.3. Feedback Bits Allocation for Maximizing the Minimum SINR

In the GBR services, we want to increase the minimum SINR in the secondary network in order to increase the data rate of the SU with the worst channel state. Hence, the problem of allocating feedback bits among multiple PUs is to maximize the minimum SINR in the secondary network while keeping the average inter-network interference below a certain value under the limited number of feedback bits for PUs, as follows:

(29) $$\begin{array}{cc} \max_{B_{k}\in R^{+}} & \min_{k\in K,m\in M_{k}}\;{\tilde{\gamma }}_{m}=\frac{{\hat{P}}_{k}\alpha_{k,m}N_{t}}{\sigma_{m}^{2}+{\hat{I}}_{k,m}+{\hat{I}}_{\bar{k},m}} \end{array}$$

(30) $$\begin{array}{cc} s.t. & \sum_{k\in K}B_{k}\leq B^{T}, \end{array}$$

where ${\hat{P}}_{k}$ is the upper bound of the transmit power of ST *k* from (15) and therefore ${\tilde{\gamma }}_{m}$ is the lower bound of ${\bar{\gamma }}_{m}$ of (23).

To provide an explicit solution with a low complexity for the above optimization problem in (29), we consider a suboptimal problem. The equivalent problem in (29) can be expressed as follows:

$$\begin{array}{c} \max_{B_{k}\in R^{+}}\;\min_{k\in K,\;m\in M_{k}}{\tilde{\gamma }}_{m} \end{array}$$

(31) $$\begin{array}{cc} = & \min_{B_{k}\in R^{+}}\;\max_{k\in K,\;m\in M_{k}}\frac{1}{{\tilde{\gamma }}_{m}} \end{array}$$

(32) $$\begin{array}{cc} = & \min_{B_{k}\in R^{+}}\;\max_{k\in K}\;c_{k}2^{\frac{-B_{k}}{L(N_{t}-1)}}, \end{array}$$

where a positive constant of $c_{k}$ is, for ∀k∈K,

(33) $$\begin{array}{c} c_{k}=\max_{m\in M_{k}}\;\;\frac{MN(\sigma_{m}^{2}+{\hat{I}}_{k,m}+{\hat{I}}_{\bar{k},m})}{I_{th}\alpha_{k,m}N_{t}}{\left(\prod_{l\in L}\alpha_{k,l}\right)}^{1/L}. \end{array}$$

Additionally, we have

(34) $$\begin{array}{ccc} \max_{k\in K}\;c_{k}2^{\frac{-B_{k}}{L(N_{t}-1)}} & = & \lim_{\tau \to \infty }{\left\{\sum_{k\in K}{\left(c_{k}2^{\frac{-B_{k}}{L(N_{t}-1)}}\right)}^{\tau }\right\}}^{\frac{1}{\tau }} \end{array}$$

(35) $$\begin{array}{cc} \geq & {\left\{\sum_{k\in K}{\left(c_{k}2^{\frac{-B_{k}}{L(N_{t}-1)}}\right)}^{\tau }\right\}}^{\frac{1}{\tau }}. \end{array}$$

Hence, for a large positive integer τ, we can approximate the optimization problem in (29) as follows:

(36) $$\begin{array}{cc} \min_{B_{k}\in R^{+}} & {\left\{\sum_{k\in K}{\left(c_{k}2^{\frac{-B_{k}}{L(N_{t}-1)}}\right)}^{\tau }\right\}}^{\frac{1}{\tau }}\\ s.t. & \sum_{k\in K}B_{k}\leq B^{T}. \end{array}$$

However, for a finite value of τ, the optimization problem in (36) is suboptimal to that of (29) because of (35).

The optimization problem in (36) is convex since τ is a constant and the inside term is convex on $B_{k}$. The Lagrangian function of (36) is given by

(37) $$\begin{array}{ccc} L(B_{k},\lambda ) & = & {\left\{\sum_{k\in K}{\left(c_{k}2^{\frac{-B_{k}}{L(N_{t}-1)}}\right)}^{\tau }\right\}}^{\frac{1}{\tau }}+\;\lambda \left(\sum_{k\in K}B_{k}-B^{T}\right), \end{array}$$

where λ is the Lagrangian multiplier. Hence, the suboptimal solution of the optimization problem in (36) should meet the Karush-Kuhun-Tucker (KKT) conditions as follows:

(38) $$\begin{array}{c} \frac{\partial L(B_{k},\lambda )}{\partial B_{k}}=-\omega c_{k}^{\tau }2^{\frac{-\tau B_{k}}{L(N_{t}-1)}}+\lambda =0,\;\forall k, \end{array}$$

where a positive constant of ω is

(39) $$\begin{array}{c} \omega =\frac{\ln(2)}{L(N_{t}-1)}{\left\{\sum_{k\in K}{\left(c_{k}2^{\frac{-B_{k}}{L(N_{t}-1)}}\right)}^{\tau }\right\}}^{\frac{1}{\tau }-1}. \end{array}$$

Additionally, we have

(40) $$\begin{array}{c} \frac{\partial L(B_{k},\lambda )}{\partial \lambda }=\sum_{k\in K}B_{k}-B^{T}=0. \end{array}$$

By substituting (39) into (40), we obtain the suboptimal solution as follows:

(41) $$\begin{array}{ccc} B_{k}^{*} & = & {\left\lfloor B^{T},\;\;\frac{B^{T}}{K}+L\left(N_{t}-1\right)\times \log_{2}\left(\frac{c_{k}}{\prod_{j\in K}{\left(c_{j}\right)}^{1/K}}\right)\right\rfloor }^{+},\;\;\forall k\in K. \end{array}$$

From (41), we can know that as the value of $c_{k}$ increases, the CDI should be more accurate in order to increase the minimum data rate of the SUs.

### 4.4. Feedback Bits Allocation for the Sum Rate Maximization with GBR Services

The proposed scheme aims to increase the sum rate of SUs while guaranteeing the minimum data rate of SUs. Hence, the optimization problem of the feedback bits allocation can be formulated as follows:

(42) $$\begin{array}{cc} \max_{B_{k}\in R^{+}} & \sum_{k\in K}\sum_{m\in M_{k}}\log_{2}\left(1+{\tilde{\gamma }}_{m}\right)\\ s.t. & \left(C1\right)\;\;{\tilde{\gamma }}_{m}\geq \gamma_{\min},\;\forall m\in M_{k},\\ & \left(C2\right)\;\;\sum_{k\in K}B_{k}\leq B^{T}, \end{array}$$

where ${\tilde{\gamma }}_{m}$, which is the lower bound of the SINR of SU *m*, is obtained from (29).

A continuous relaxation technique can by applied to the integer constraint of the number of feedback bits to derive a closed form solution. The constraint of (C1) means the secondary network should guarantee the minimum SINR, $\gamma_{\min}$, of SUs, in order to provide the GBR services for SUs. However, if the total number of feedback bits, $B^{T}$, is not enough, the outage probability may occur. From (27), we can replace the constraint of (C1) with $B_{k}\geq {\tilde{B}}_{k,m}$. That is, the number of feedback bits for ST *k* should be greater than or equal to ${\tilde{B}}_{k,m}$ in order to guarantee the minimum data rate of SU *m* served by ST *k*. Moreover, the constraint of (C2) means the total number of feedback bits is limited. We rewrite the integrated optimization problem as follows:

(43) $$\begin{array}{cc} \max_{B_{k}\in R^{+}} & \sum_{k\in K}\sum_{m\in M_{k}}\log_{2}\left(1+{\tilde{\gamma }}_{m}\right)\\ s.t. & \left(C1\right)\;\;B_{k}\geq {\tilde{B}}_{k,m},\;\forall m\in M_{k},\\ & \left(C2\right)\;\;\sum_{k\in K}B_{k}\leq B^{T}. \end{array}$$

From (43), we find a tradeoff between ${\tilde{B}}_{k,m}\leq B_{k}$ and $\sum_{k\in K}B_{k}\leq B^{T}$. That is, if the total number feedback bits is not enough, i.e., $\sum_{k\in K}\max_{m\in M_{k}}{\tilde{B}}_{k,m}>B^{T}$, the outage probability may occur and the minimum data rate of some SUs may not be guaranteed. We aim to reduce the outage probability of the GBR services for SUs while increasing the average sum rate of SUs. To solve the optimization problem of (43), we take a phased approach. First, in order to reduce the outage probability when the total number feedback bits is not enough, we find a pair of (SU *m* and ST *k*) with the minimum value of ${\tilde{B}}_{k,m}$ to reduce the required number of feedback bits. Then, we dynamically allocate the remaining feedback bits to STs in order to increase the sum rate of SUs. We develop a heuristic algorithm to allocate feedback bits to PUs, as shown in Algorithm 1.
**Algorithm 1** Proposed feedback bits allocation algorithm/* Initialization */**1:** Initialize $B_{k,\mathrm{req}}\leftarrow \max_{m\in M_{k}}{\tilde{B}}_{k,m}$ for ∀k**2:** Set $B_{k,\min}\leftarrow 0$ for ∀k  /* Feedback bits allocation for increasing the min SINR */**3:** **if**$\sum_{k\in K}B_{k,\mathrm{req}}<B^{T}$**then****4:**     update $B_{k,\min}\leftarrow B_{k,\mathrm{req}}$ for ∀k**5:** **else****6:**    **while** $\sum_{k\in K}B_{k,\min}<B^{T}$ **do****7:**        Update $k^{*},m^{*}\leftarrow \arg\min_{k\in K,m\in M_{k}}{\tilde{B}}_{k,m}$**8:**        **if** ${\tilde{B}}_{k^{*},m^{*}}+\sum_{k\in K,k\neq k^{*}}B_{k,\min}<B^{T}$ **then****9:**           Update $B_{k^{*},\min}\leftarrow {\tilde{B}}_{k^{*},m^{*}}$**10:**         Update $M_{k^{*}}\leftarrow M_{k^{*}}\setminus \left\{m^{*}\right\}$**11:**        **else****12:**           Update $B_{k^{*},\min}\leftarrow B^{T}-\sum_{k\in K,k\neq k^{*}}B_{k,\min}$**13:**        **end if****14:**    **end while****15:** **end if**  /* Feedback bits allocation for increasing the sum rate of SUs when K=2*/**16:** **Set**$B_{R}\leftarrow B^{T}-\sum_{k\in K}B_{k,\min}$**17:** Initialize $B^{\mathrm{LB}}\leftarrow 0$ and $B^{\mathrm{UB}}\leftarrow B_{R}$**18:** **if**$B_{R}>0$**then****19:**    Set $B_{1}\leftarrow {\left\lfloor B^{\mathrm{LB}}+B^{\mathrm{UB}}\right\rfloor }^{+}$ and $B_{2}\leftarrow B_{R}-B_{1}$**20:**    **while** $B^{\mathrm{LB}}\neq B_{1}$ and $B^{\mathrm{UB}}\neq B_{1}$ **do****21:**        **if then** $\sum_{k\in K}\sum_{m\in M_{k}}\frac{\partial {\tilde{R}}_{m}}{\partial B_{k}}>0$**22:**           $B^{\mathrm{LB}}\leftarrow B_{1}$**23:**        **else****24:**           $B^{\mathrm{UB}}\leftarrow B_{1}$**25:**        **end if****26:**        Update $B_{1}\leftarrow {\left\lfloor B^{\mathrm{LB}}+B^{\mathrm{UB}}\right\rfloor }^{+}$**27:**    **end while****28:** **end if****29:** **return**$B_{k,\min}$ for ∀k

The operation of Algorithm 1 is as follows: First, we set two initialization parameters, $B_{k,\mathrm{req}}$ and $B_{k,\min}$, where $B_{k,\mathrm{req}}$ is the number of required feedback bits between ST *k* and PUs to guarantee the minimum data rate for SUs served by ST *k*; and $B_{k,\min}$ is the number of temporarily allocated feedback bits between ST *k* and PUs. From *Step 3* to *Step 15*, we gradually increase the value of $B_{k,\min}$ by using (27) in order to increase the number of SUs who receive the GBR services. If $\sum_{k\in K}B_{k,\mathrm{req}}>B^{T}$, the minimum data rate of some SUs may not be guaranteed due to the limitation of the total number of feedback bits; and the algorithm terminates at *Step 15*. Otherwise, i.e., if $\sum_{k\in K}B_{k,\mathrm{req}}<B^{T}$, the minimum data rate of all the SUs will be guaranteed. From *Step 16* to *Step 29*, the algorithm allocates the remaining feedback bits, $B_{R}=B^{T}-\sum_{k\in K}B_{k,\min}$, to STs in order to maximize the average sum rate of SUs. In Algorithm 1, we assume there are two STs, K={1,2}. In line 21 of the algorithm, ${\tilde{R}}_{m}$, which denotes the lower bound of the average data rate of SU *m*, is given by ${\tilde{R}}_{m}=\log_{2}\left(1+{\tilde{\gamma }}_{m}\right)$, where ${\tilde{\gamma }}_{m}$, which is a function of $B_{k}$, is obtained from (29).

## 5. Numerical Results

**Figure 2:**
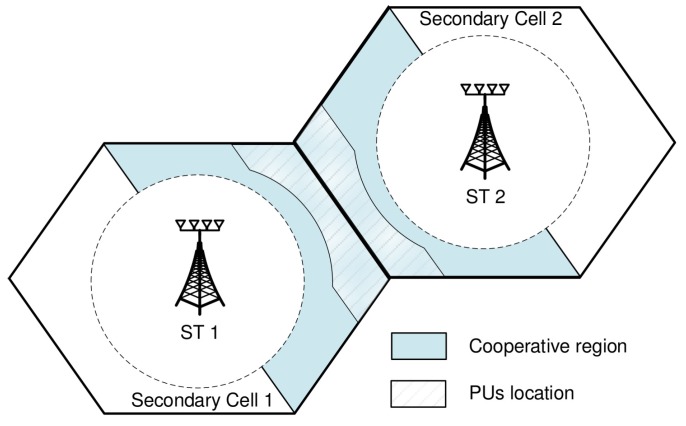
A cooperative region in the simulation.

An underlay CR network with L=2, M=2, K=2, and $N_{t}=6$ is considered. For all scenarios, $P_{\max}$ is set as $P_{\max}/\sigma_{m}^{2}=0$ dB for ∀m, and the average inter-network interference constraint is $I_{th}=0$ dB. Additionally, we assume that the path loss coefficient is 3.8 and the standard deviation for large-scale shadowing is $\sigma_{S}=8$ dB. We uniformly distributed the location of SUs according to the coordination area of [23]. As shown in Figure 2, all SUs lie in the region 0.325$\leq d_{k,m}\leq$0.5, where $d_{k,m}$ is the normalized distance from ST *k* to SU *m*. Meanwhile, all PUs are distributed in the region 0.35$\leq d_{k,l}\leq$0.65, where $d_{k,l}$ is the normalized distance from ST *k* to PU *l*. The numerical results are obtained by taking an average of 50,000 drop events of PUs and SUs. Here, the drop event means a simulation event that SUs randomly drop in the region 0.325$\leq d_{k,m}\leq$0.5 and PUs randomly drop in the region 0.35$\leq d_{k,l}\leq$0.65; and we ran $N_{sim}=50,000$ simulations.

For the performance comparison, we consider two conventional schemes, the equal feedback bits allocation (EFA) and the adaptive feedback bits allocation (AFA). In the EFA scheme, the number of feedback bits allocated to each SU is the same and the number of feedback bits allocated each PU is also the same, i.e., $b_{k,m}=b^{T}/K$ for ∀m and $B_{k,l}=B^{T}/LK$ for ∀k,l. In the AFA scheme, the number of feedback bits is adaptively allocated to SUs according to (20) in Section 3 in order to minimize the average rate loss while the number of feedback bits allocated to each PU is the same. Because the previous work of [17] considers a single secondary cell, K = 1, the proposed scheme of [17] belongs to the AFA scheme. Then, in the conventional EFA and AFA schemes, the transmit power of ST *k* is given by (11).

**Figure 3:**
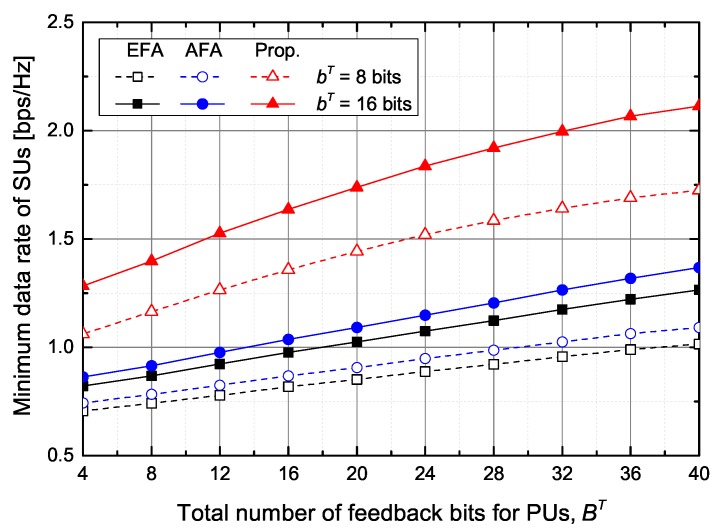
The minimum data rate of SUs versus the total number of feedback bits for PUs.

**Figure 4:**
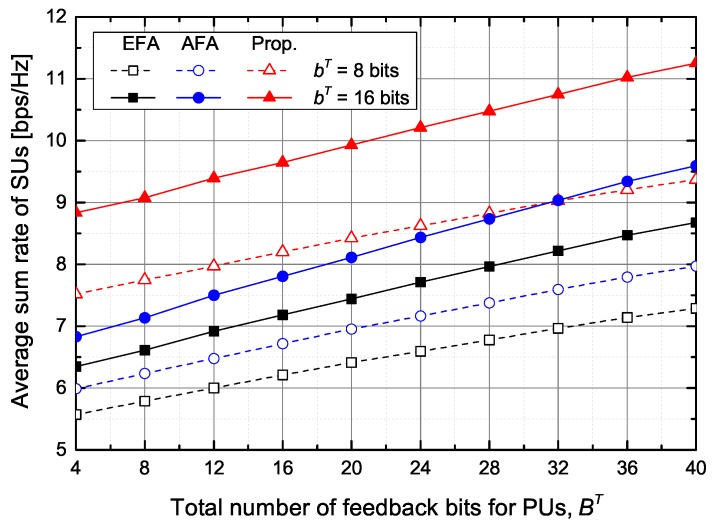
The average sum rate of SUs versus the total number of feedback bits for PUs.

**Figure 5:**
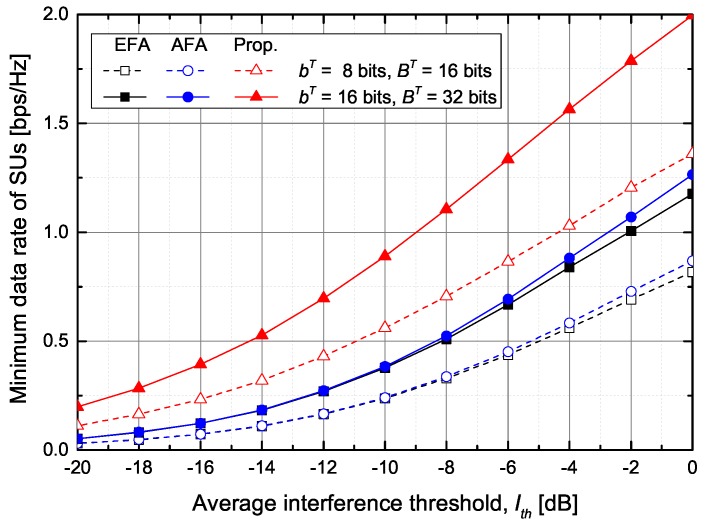
The minimum data rate of SUs versus the average inter-network interference threshold.

**Figure 6:**
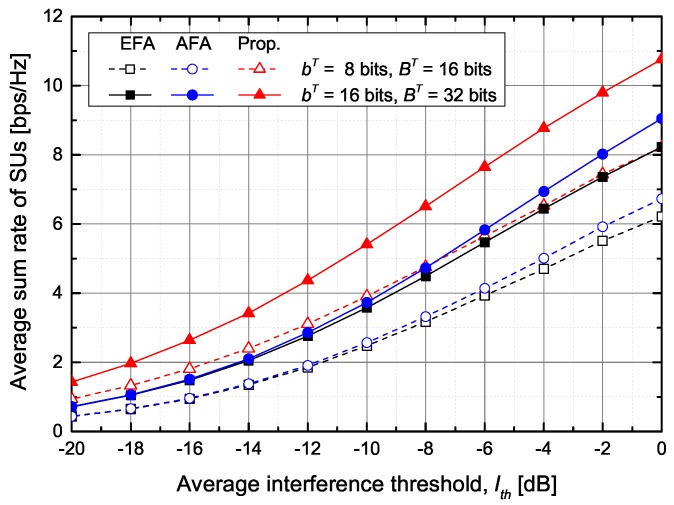
The average sum rate of SUs versus the average inter-network interference threshold.

First, from Figure 3, Figure 4, Figure 5 and Figure 6, we evaluate the performance of the feedback bits allocation scheme proposed in Section 4.3, in terms of maximizing the minimum SINR among SUs. Figure 3 shows the supported minimum data rate of SUs according to the number of feedback bits for PUs and SUs under the average inter-network interference constraint at PUs. As the number of feedback bits for PUs, $B^{T}$, increases, the ST can increase the transmit power while satisfying the interference constraint. Hence, the minimum data rate of SUs increases with the increase of $B^{T}$. The proposed scheme outperforms the conventional EFA and AFA schemes. In particular, when $B^{T}=20$ bits and $b^{T}=8$ bits, the proposed scheme increases the minimum data rate of SUs by about 69.5% and 59.1%, respectively, in comparison with the EFA and AFA schemes. Although the AFA scheme adaptively allocates the feedback bits to SUs, it slightly increases the minimum data rate of SUs compared with the EFA scheme because the conventional AFA scheme does not adaptively allocate the feedback bits to PUs.

Figure 4 shows the average sum rate of SUs according to the number of feedback bits for PUs and SUs under the average inter-network interference constraint at PUs. As the number of feedback bits for PUs, $B^{T}$, increases, the average sum rate of SUs increases thanks to the increase of the transmit power of STs. Similarly, as the number of feedback bits for SUs, $b^{T}$, increases, the average sum rate of SUs also increases because IUI and ICI decrease due to more accurate beamforming vectors.

Figure 5 shows the supported minimum data rate of SUs according to the average inter-network interference threshold. As the average interference constraint is relaxed, the minimum data rate of the SUs increases because it increases the allowable transmit power of the ST. The conventional schemes show almost the same minimum data rate of SUs. When $B^{T}=32$ bits and $b^{T}=16$ bits, the proposed scheme increases the minimum data rate of SUs by about 116.7% and 110.8%, respectively, in comparison with the EFA and AFA schemes. In particular, when the minimum data rate is 1 bps/Hz, the average interference from the ST to PUs becomes about −2 dB, −2.5 dB, and −9 dB in the EFA, AFA, and proposed schemes, respectively.

Figure 6 shows the average sum rate of SUs according to the average inter-network interference threshold. The proposed scheme shows that the average sum rate of SUs is higher than that of the conventional schemes. For example, when $B^{T}=32$ bits and $b^{T}=16$ bits, the proposed scheme improves the average sum rate of SUs by about 45.0% and 37.5%, respectively, in comparison with the EFA and AFA schemes.

Although the feedback bits allocation scheme proposed in Section 4.3 increases the minimum data rate of the SUs, it may not guarantee the minimum data rate of SUs because of the limited number of feedback bits. Hence, we need to determine if the number of feedback bits is sufficient to meet the minimum data rate constraint of SUs, by using (28) which is the scaling law of the number of feedback bits.

**Figure 7:**
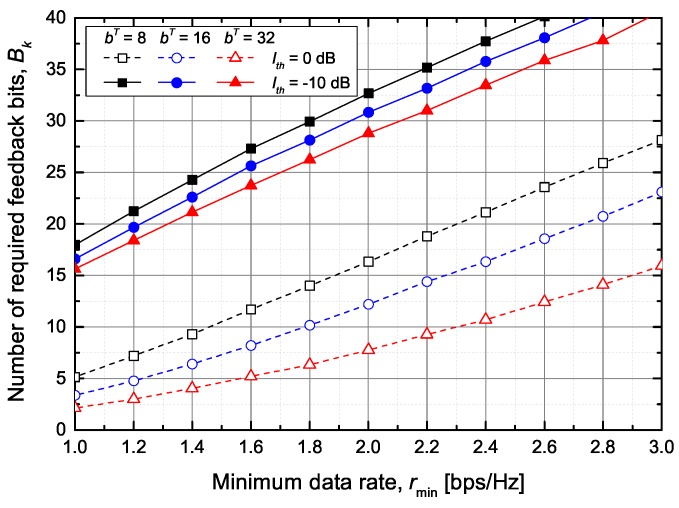
The average number of required feedback bits between ST *k* and PUs.

Figure 7 shows the average number of required feedback bits between ST *k* and PUs to guarantee the minimum data rate of SU $m\in M_{k}$ when the total number of feedback bits is fixed to $B^{T}=40$ bits. As expected, the number of required feedback bits $B_{k}$ increases as the value of $r_{\min}$ increases. However, even if the value of $B_{k}$ exceeds the total number of feedback bits, the minimum bit rate of SU *m* may not be guaranteed. For example, consider $I_{th}=-10$ dB and $b^{T}=8$ bits. If the value of $r_{\min}$ exceeds 2.6 bps/Hz, the secondary network does not guarantee the minimum data rate even for a single SU. Furthermore, if the sum of $B_{k}$ exceeds $B^{T}$, the secondary network will not meet the minimum data rate of multiple SUs. Accordingly, we aim to reduce the SU’s outage probability while maximizing the sum rate of SUs by using the proposed feedback bits allocation scheme described in Section 4.4.

The performance of the proposed scheme is compared with three other schemes, such as the conventional EFA and AFA schemes and the AFA2 scheme. Here, the AFA2 scheme adaptively allocates the feedback bits to PUs according to (12) in Section 2.3. Table 1 summarizes the feedback bits allocation strategy.

**Figure 8:**
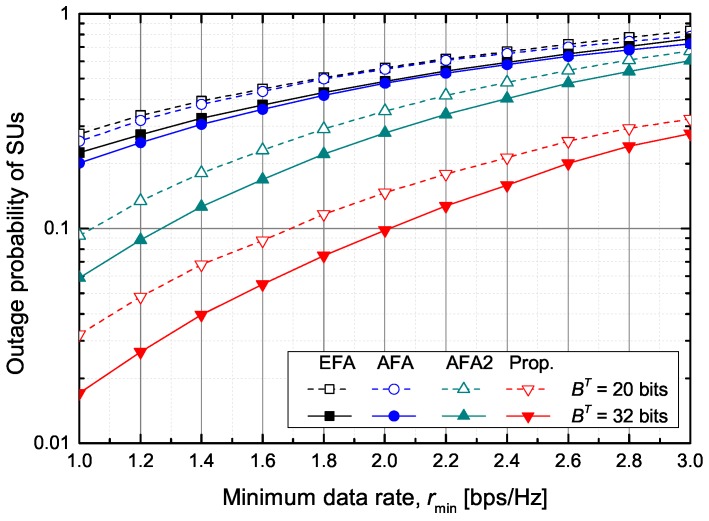
The outage probability versus the minimum data rate.

Figure 8 shows the outage probability of SUs according to the required minimum data rate of SUs when $I_{th}=0$ dB and $b^{T}=16$ bits. The outage probability is calculated by $P_{out}=M_{out}/\left(N_{sim}\cdot K\cdot M\right)$, where $M_{out}$ is the number of SUs that cannot attain the minimum data rate during $N_{sim}$ simulations, *K* is the number of secondary cells in the secondary network, and *M* is the number of SUs in each secondary cell. The EFA and AFA schemes show nearly the same outage probability. That is, if the number of feedback bits allocated to the PUs is the same, it is difficult to guarantee the minimum data rate of SUs because the transmit power of STs cannot be adaptively adjusted. The AFA2 scheme outperforms the EFA and AFA schemes because it dynamically sets the value of $B_{k,l}$ under the fixed value of $B_{k}=B^{T}/K$. The proposed scheme shows the best performance. In particular, when $B^{T}=32$ bits and the outage probability should be below 10%, the minimum guaranteed data rate is about 2.0 bps/Hz in the proposed scheme and about 1.25 bps/Hz in the AFA2 scheme, respectively.

**Figure 9:**
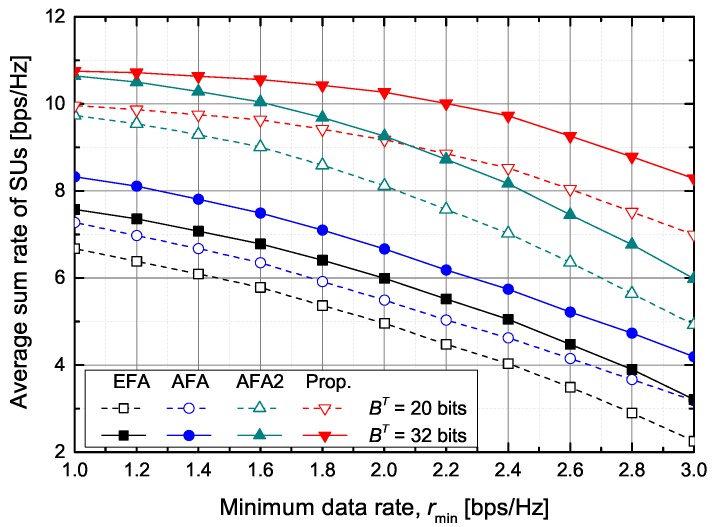
The achievable average sum rate of SUs versus the minimum data rate.

Figure 9 shows the average sum rate of SUs according to the required minimum data rate of SUs when $I_{th}=0$ dB and $b^{T}=16$ bits. The proposed scheme outperforms the other schemes, the EFA, AFA, and AFA2 schemes. When $r_{\min}=2$ bps/Hz and $B^{T}=32$ bits, the proposed scheme increases the achievable average sum rate of SUs by about 71.2%, 54.0%, and 10.9%, respectively, in comparison with the EFA, AFA, and AFA2 schemes.

## 6. Conclusions

This paper proposed a feedback bits allocation scheme for the CR network that provides the GBR services for SUs. We first developed the optimization problem that maximizes the minimum data rate of SUs with the fixed number of feedback bits. We found the suboptimal number of feedback bits between STs and PUs as a closed-form by applying a continuous relaxation technique for the integer constraint. On the basis of the closed form of the achievable average data rate of SUs, we derived the required number of feedback bits, between the ST and PUs, needed to guarantee the minimum data rate of SUs. Moreover, we developed the integrated optimization problem that maximizes the sum rate of SUs while reducing the outage probability of SUs. The proposed feedback bits allocation scheme increases the minimum data rate of SUs, without sacrificing the average sum rate of SUs, in comparison with the conventional schemes.

## Figures and Tables

**Table 1:** Comparison of the feedback bits allocation strategy.

Parameter	EFA	AFA [17]	AFA2	Proposed
$B_{k}$	Fixed	Fixed	Fixed	Adaptive
$B_{k,l}$	Fixed	Fixed	Adaptive	Adaptive
$b_{k,m}$	Fixed	Adaptive	Adaptive	Adaptive
